# Detecting Emotional Signals of Relationship Well-Being Using AI: A Cross-Sectional Comparison of Transformer-Based and Lexicon-Based Models for Couples Therapy Research

**DOI:** 10.2196/94097

**Published:** 2026-11-09

**Authors:** Alejandro Saavedra-Roa, Claudia Pineda-Marin, Pablo Vallejo-Medina, Adrián Montesano

**Affiliations:** 1 Faculty of Psychology and Education Sciences, Universitat Oberta de Catalunya, Rambla del Poblenou, 156, Barcelona, Catalonia, 08018, Spain, 1 934505225; 2 Department of Psychology, Fundación Universitaria Konrad Lorenz, Bogotá DC, Colombia

**Keywords:** artificial intelligence, AI, natural language processing, emotion detection, transformer models, lexicon-based methods, couple flourishing

## Abstract

**Background:**

Natural language processing and AI models are increasingly used to extract emotional signals from textual data; however, systematic validation of different computational paradigms in emotionally complex relational contexts remains limited. Such approaches may contribute to digital health research, particularly in the fields of digital mental health and couples therapy, where understanding emotional expressions within romantic relationships is especially relevant.

**Objective:**

This study aimed to compare a lexicon-based approach (NRC Emotion Lexicon) and a transformer-based model (Pysentimiento) in detecting affective patterns within spontaneous romantic relationship narratives and to evaluate their criterion-related associations with a theory-driven measure of relational well-being.

**Methods:**

In this cross-sectional exploratory study, 112 Spanish adults completed the Couple Flourishing Measure (CFM) and provided an open-ended description of their romantic relationship. Narratives were analyzed using both computational approaches to generate emotion and sentiment indices. Pearson correlations were computed between model outputs and CFM scores. Steiger Z tests were conducted to compare dependent correlations across models.

**Results:**

Both approaches showed significant associations with couple flourishing, although the transformer-based model yielded stronger correlations for several affective dimensions, particularly positive sentiment (*r*=0.66, 95% CI 0.54-0.76) and negative sentiment (*r*=−0.74, 95% CI −0.82 to −0.65). The lexicon-based approach showed smaller associations, primarily for negative emotions. Steiger Z tests identified significant between-model differences for disgust (*P*=.04), joy (*P*=.01), sadness (*P*=.004), surprise (*P*=.03), and positive and negative sentiment (both *P*<.001), but not for anger or fear.

**Conclusions:**

In this sample, Pysentimiento showed stronger associations with couple flourishing than the NRC Emotion Lexicon across several affective dimensions, although differences were not consistent across all categories. These findings support the value of theory-driven external criteria for evaluating computational emotion detection in relational narratives and underscore the importance of context-sensitive interpretation when applying AI tools in psychologically sensitive domains. These findings also highlight the potential relevance of computational emotion analysis for digital health research, particularly in digital mental health and couples therapy research, although further validation in clinical settings is needed before clinical implementation.

## Introduction

Natural language processing (NLP) has become an increasingly relevant methodological frontier in the social and behavioral sciences, allowing researchers to explore language as a dynamic expression of emotion, cognition, and relational experience [1-3]. Recent advances in AI, particularly large-scale language models, have accelerated the capacity to model affective meaning from natural discourse. Within the field of family and couple therapy research, NLP offers novel opportunities to move beyond traditional self-report measures toward the automated analysis of natural discourse, a shift that may deepen the theoretical understanding of affective dynamics and relational well-being.

One of the most significant advances in NLP is the emergence of the transformer architecture [4], which enables large-scale modeling of textual meaning through contextual attention mechanisms. Transformers have demonstrated remarkable performance across linguistic and psychological tasks, overcoming many of the limitations of earlier sequential models [5-7]. For example, Steinbrenner et al [8] used NLP to predict anxiety severity from psychotherapy transcripts, illustrating the potential clinical applicability of these methods. However, transformer-based models also have architectural limitations that warrant caution when interpreting their outputs, particularly when applied to emotionally complex language in couple or family contexts [6]. Despite these advances, relatively few studies have systematically compared contextual AI-based models and lexicon-based approaches using theory-driven external validation within relational or clinically sensitive domains.

Beyond transformer architectures, emotion detection in textual data has traditionally relied on lexicon-based approaches in which words are compared with predefined dictionaries that associate them with certain emotions or sentiment categories [9,10]. These resources, such as the NRC Emotion Lexicon, offer transparent and easily interpretable mappings between linguistic units and affective constructs and have been widely used in psychological, social, and computational research for over a decade [11,12]. However, lexical models have significant limitations in capturing contextual meaning, polysemy, irony, or relational nuances in discourse [13]. In contrast, deep learning models, such as transformers, infer emotional signals from contextual representations (contextual embeddings), which allows for the identification of more subtle and context-dependent affective patterns in natural language [4,14]. Thus, lexicon-based and neural network–based methods represent complementary paradigms within computational emotion analysis, each with distinct strengths and limitations. Analyzing how these approaches work when applied to narratives about romantic relationships offers a valuable methodological opportunity to evaluate how different NLP frameworks conceptualize affective meaning in relational contexts.

Among the available transformer-based tools, Pysentimiento [15-17] stands out for its open access, multilingual design, and capacity to perform several affective tasks, including sentiment and emotion detection, irony identification, and hate speech analysis. Preliminary applications of Pysentimiento have shown promise in detecting emotional patterns in diverse social domains, such as news consumption (ie, linguistic descriptors of physiological responses produced by fake news), education, and gastronomy [18-20]. However, its potential contribution to relationship and family therapy studies remains unexplored.

In the context of couple relationships, affective states such as joy, sadness, anger, and fear are central to both relationship quality and emotional regulation processes [21,22]. Emotional communication patterns help couples construct shared meanings, maintain intimacy, and recover from conflict [23-25]. Constructs such as couple flourishing integrate these affective dimensions by conceptualizing relational well-being as a dynamic state of mutual growth, compassion, and positive engagement [26]. However, existing tools for assessing affective experiences, mostly self-report instruments, may fall short in capturing the nuance and spontaneity of natural relational language, and their use often depends on clinicians’ subjective interpretation or training [27,28]. Recent work in psychotherapy research has begun validating sentiment-analysis approaches as tools for understanding in-session emotional processes [29], with more recent large-scale validation studies also contributing to this research [30]. These developments highlight the growing relevance of NLP-based methods for capturing affective dynamics in clinical settings.

Despite the relevance of these affective processes for relational functioning, their assessment is particularly challenging in therapeutic contexts, where emotional expression and dyadic regulation are the central mechanisms of change [31]. In couple interventions, much of the therapeutic work is carried out through narrative, dialogue, and meaning-making; therefore, the linguistic and affective qualities of the couple’s discourse represent a critical source of clinical information [32]. However, traditional observational coding systems tend to be time-consuming, require extensive training, and have limitations in capturing subtle changes or relational microprocesses that emerge during therapeutic conversations [33]. In turn, self-report measures may not reflect the moment-to-moment affective shifts that arise in the dialogue between couple members [27]. In this sense, methodological innovations capable of extracting emotional signals directly from natural language offer a promising avenue for advancing research on processes in couples therapy, allowing for the identification of relational patterns, affective exchanges, and possible indicators of therapeutic progress [34]. NLP-based approaches, especially those supported by context-sensitive architectures, can deepen our understanding of dyadic and systemic processes as they unfold in intervention settings.

To address these challenges, the present study proposes a methodological comparison at the intersection of couple relationships and computational linguistics, contrasting 2 NLP-based approaches to emotional assessment: a transformer-based model (Pysentimiento) and a lexicon-based model (NRC Emotion Lexicon). Specifically, this study provides a comparative evaluation of 2 computational paradigms for emotion detection using theory-driven external validation against a validated measure of relational well-being. By examining their associations with the Couple Flourishing Measure (CFM) [26] in a Spanish-speaking sample, we aimed to evaluate how different NLP paradigms capture affective meanings in intimate partners’ narratives. By situating this comparison within a clinically sensitive relational domain, the study contributes to emerging discussions on the responsible and methodologically rigorous deployment of AI-driven language models in mental health and relational research. Accordingly, this study aimed to (1) examine the associations between NRC Emotion Lexicon– and Pysentimiento-derived affective indices and couple flourishing, and (2) determine whether the strength of these associations differed between the 2 approaches.

## Methods

### Participants

A total of 112 adults residing in Spain participated in this exploratory cross-sectional study. Participants were recruited through nonprobability convenience sampling, given the exploratory and methodological focus of the research. All participants had been in a romantic relationship for at least 6 months at the time of the study. No a priori sample size calculation was performed due to the exploratory nature of the study.

### Instruments

#### Psychosexual and Sociodemographic Information

An ad hoc questionnaire was designed to collect psychosexual and sociodemographic information from the participants. This instrument allowed us to obtain data on age, place of residence during the study, gender, duration of the couple’s relationship, and sexual orientation according to Kinsey et al [35].

#### CFM

In this study, we used the version validated in Spain by Saavedra-Roa et al [36,37]. It is a 16-item unidimensional instrument that is answered individually and assesses couple flourishing on a 7-point visual analog Likert scale, where 1=distressed relationship, 4=satisfied relationship, and 7=flourishing relationship. An example item is “Quality time with my partner alone is...,” where 1=distressed, 4=satisfied, and 7=flourishing. The scores ranged from 16 to 112, with higher scores indicating higher levels of couple flourishing. This instrument has been shown to have high reliability (α=.94), which was α=.95 for this study.

#### Affective-Sexual Relationship Disclosure

A single open-ended question was used to capture the participants’ natural discursive responses. Individuals were asked to answer the question “How would you describe your couple relationship?” This prompt was chosen to encourage spontaneous and emotionally rich descriptions of the relationship, thus providing linguistic material suitable for subsequent computational emotion analysis. The narratives generated through this item served as the textual dataset for both NLP analyses (refer to the Data Analysis section).

### Procedure

All participants completed an online survey administered via Qualtrics, which included sociodemographic and psychosexual information, the CFM, and an open-ended question regarding their perceptions of their affective-sexual relationship. The sampling was conducted between June 2023 and June 2024. Recruitment was conducted primarily through dissemination on social media platforms. Participants accessed the study via a link, provided digital informed consent, and completed the survey anonymously. The average response time of the battery was 28.08 minutes.

### Data Analysis

#### Overview

All the narratives obtained from the open-ended question were processed in Spanish. Text processing differed between the 2 NLP approaches. For the lexicon-based approach, narratives were tokenized into words for comparison with lexicon entries, whereas the transformer-based approach was applied to the original, unprocessed responses. Subsequently, 2 NLP approaches were used to extract affective information from the narratives: a model based on the transformer architecture (Pysentimiento) and a lexicon-based model (NRC Emotion Lexicon, multilingual version 2022).

#### Transformer-Based Analysis

Indicators of emotional polarity (ie, positive, negative, and neutral) and 6 basic emotions (ie, joy, sadness, anger, disgust, fear, and surprise) were obtained using Pysentimiento [15-17], an open-source model implemented in Python and based on the transformer architecture with support for Spanish-language text. For each narrative, the tool generated probabilistic scores ranging from 0 to 1, which estimated the presence of each emotion or sentiment dimension. Pysentimiento was applied to each participant’s complete, unprocessed response, and the resulting scores were retained at the participant level.

#### Lexicon-Based Analysis

Narratives were also analyzed using the Spanish version of the NRC Emotion Lexicon [12], a lexical resource that associates more than 14,000 lemmas with 8 emotions (ie, anger, fear, anticipation, trust, surprise, sadness, joy, and disgust) and 2 sentiment categories (ie, positive and negative). Each narrative was processed using the *syuzhet* package in R (version 4.4.1; R Foundation for Statistical Computing) [38], with words matched against lexicon entries. Lexicon matches were aggregated within each complete response to obtain participant-level scores for the 8 emotions and 2 sentiment categories. The unit of analysis was the complete open-ended response of each participant (n=112), rather than individual words or sentences. This unit was selected because the CFM was assessed at the participant level and the open-ended question elicited an overall description of the romantic relationship. Moreover, the relatively short narratives made further subdivision into sentences less appropriate for the study objectives.

### Statistical Analysis

Correlations between the total scores on the CFM and the emotional and sentiment indices generated by both NLP models were examined using Pearson correlation coefficients (*r*). For each participant, the scores generated by both models were matched with their corresponding CFM total score, which served as a common external criterion for evaluating the associations between the affective indices and relationship flourishing. To determine whether the correlations obtained using Pysentimiento differed significantly from those obtained using the NRC Emotion Lexicon, Steiger Z tests for dependent correlations were performed for the emotion and sentiment categories shared by both models. This test was selected because the correlations were derived from the same sample and shared the CFM score as a common variable, while accounting for the correlation between the corresponding model-derived indices. Categories available in only 1 model were not included in these comparisons. The 95% CIs were estimated for the reported effects. Additionally, an exploratory ordinary least squares linear regression was performed to examine the trend in CFM total scores across dominant sentiment categories. Dominant sentiment was defined as the category with the highest predicted probability from Pysentimiento, with categories coded as ordered levels (1=negative, 2=neutral, and 3=positive). Missing data were handled using available-case analysis. All analyses were carried out using R [39] in the R Studio environment (version 2023.06.1+524; Posit PBC) [40].

### Ethical Considerations

This study was approved by the Ethics Committee, which operates independently of the Faculty of Psychology and Educational Sciences. This study was conducted in accordance with the principles of the 1975 Declaration of Helsinki, as revised in 1983 by the Research Ethics Committee. All participants voluntarily agreed to participate in the study, and the ethical committee evaluated the procedure to obtain consent. This study was approved by the Ethics Committee of the university, with an approval number CE23-PF02.

## Results

### Participant Characteristics

The sample comprised 112 adults residing in Spain. Age data were available for 102 participants, with a mean age of 34.66 (SD 10.22) years. Of the 112 participants, 24.1% (n=27; mean age 34.0, SD 9.37 years) were men and 75.9% (n=85; mean age 34.85, SD 10.49 years) were cisgender women. No significant age differences were observed between genders (*t*_100_=−0.34; *P*=.73). Regarding sexual orientation, 89.3% (n=100) identified as exclusively or predominantly heterosexual, 5.4% (n=6) as exclusively or predominantly homosexual, 2.7% (n=3) reported intermediate patterns of heterosexual and homosexual attraction or contact, and 1.8% (n=2) identified as asexual. Sexual orientation data were missing for 1 (0.9%) participant.

### Associations Between the NRC Emotion Lexicon and the CFM Scores

Correlations between scores on the NRC Emotion Lexicon and the CFM were examined. The emotions of anger, disgust, fear, sadness, and the negative category showed significant negative correlations with CFM scores, although their magnitudes were low (*r*=–0.19 to *r*=–0.27). The dimensions of joy, surprise, trust, and the positive category did not show significant associations.

### Associations Between Pysentimiento and CFM Scores

Subsequently, the correlations between the scores generated by Pysentimiento and the CFM were analyzed. Compared with the NRC Emotion Lexicon, this transformer model showed stronger and more consistent associations with CFM scores. The emotions of disgust, sadness, fear, and anger showed higher negative correlations (*r*=–0.27 to *r*=–0.49). Joy, as well as the positive and negative categories, showed significant and moderate correlations, highlighting the strong association between negative (*r*=–0.74) and positive feelings (*r*=0.66) with the CFM.

### NRC Emotion Lexicon and Pysentimiento

Statistically significant differences between the models were observed for disgust, joy, sadness, surprise, and positive and negative sentiment. Pysentimiento-derived scores showed stronger associations with CFM scores for disgust, joy, sadness, and both sentiment dimensions, whereas the associations for anger and fear did not differ significantly between models. For surprise, the models showed correlations in opposite directions, with a nonsignificant positive association for NRC Emotion Lexicon and a significant negative association for Pysentimiento. Overall, these findings indicate that the transformer-based approach showed stronger criterion-related associations across several affective dimensions, although this pattern was not uniform across all categories (Table 1).

**Table 1.** Comparative correlations between NRC Emotion Lexicon and Pysentimiento emotion scores with Couple Flourishing Measure (CFM) (N=112)^a^.

Variable	NRC Emotion Lexicon, *r* (95% CI)	*P* value	Pysentimiento, *r* (95% CI)	*P* value	Steiger Z^b^	Difference (95% CI)	*P* value
Anger	–0.24 (–0.41 to –0.06)	.01	–0.36 (–0.51 to –0.19)	<.001	1.36	0.12 (–0.05 to 0.29)	.17
Anticipation	–0.04 (–0.22 to 0.15)	.68	—^c^	—	—	—	—
Disgust	–0.24 (–0.41 to –0.06)	.01	–0.42 (–0.56 to –0.26)	<.001	–2.03	0.18 (0.007 to 0.356)	.04
Fear	–0.20 (–0.37 to –0.01)	.03	–0.27 (–0.43 to –0.09)	.004	0.51	0.07 (–0.19 to 0.32)	.61
Joy	0.11 (–0.08 to 0.29)	.24	0.40 (0.23 to 0.54)	<.001	–2.48	–0.29 (–0.50 to –0.06)	.01
Sadness	–0.19 (–0.36 to –0.01)	.04	–0.49 (–0.62 to –0.33)	<.001	2.87	0.30 (0.09 to 0.49)	.004
Surprise	0.09 (–0.10 to 0.27)	.34	–0.22 (–0.39 to –0.04)	.02	2.20	0.31 (0.03 to 0.57)	.03
Trust	0.14 (–0.04 to 0.32)	.14	—	—	—	—	—
Negative	–0.27 (–0.43 to –0.09)	.004	–0.74 (–0.82 to –0.65)	<.001	5.76	0.47 (0.30 to 0.64)	<.001
Positive	0.17 (–0.01 to 0.35)	.07	0.66 (0.54 to 0.76)	<.001	–4.85	–0.49 (–0.68 to –0.29)	<.001

a *P* values were calculated from the reported Pearson correlation coefficients rounded to two decimal places and are therefore approximate.

b Steiger Z tests were used to compare overlapping dependent correlations obtained from the same participants and sharing CFM as the common criterion variable.

c Not applicable because anticipation and trust were not included in Pysentimiento's classification scheme.

### Overall Trend Between Dominant Sentiment and CFM Scores

As an additional exploratory analysis, we examined the overall relationship between the dominant sentiment identified by Pysentimiento (ie, negative, neutral, or positive) and CFM scores. A significant positive linear trend was observed (B=22.80, 95% CI 18.52-27.09; *t*_110_=10.55; *P*<.001), explaining 50.3% of the variance in CFM scores (*R*^2^=0.503). Mean CFM scores increased from 40.1 (SD 20.6; n=13) in the negative sentiment group to 75.7 (SD 18.4; n=19) in the neutral group and 90.2 (SD 13.6; n=80) in the positive group (Figure 1).

**Figure 1.**
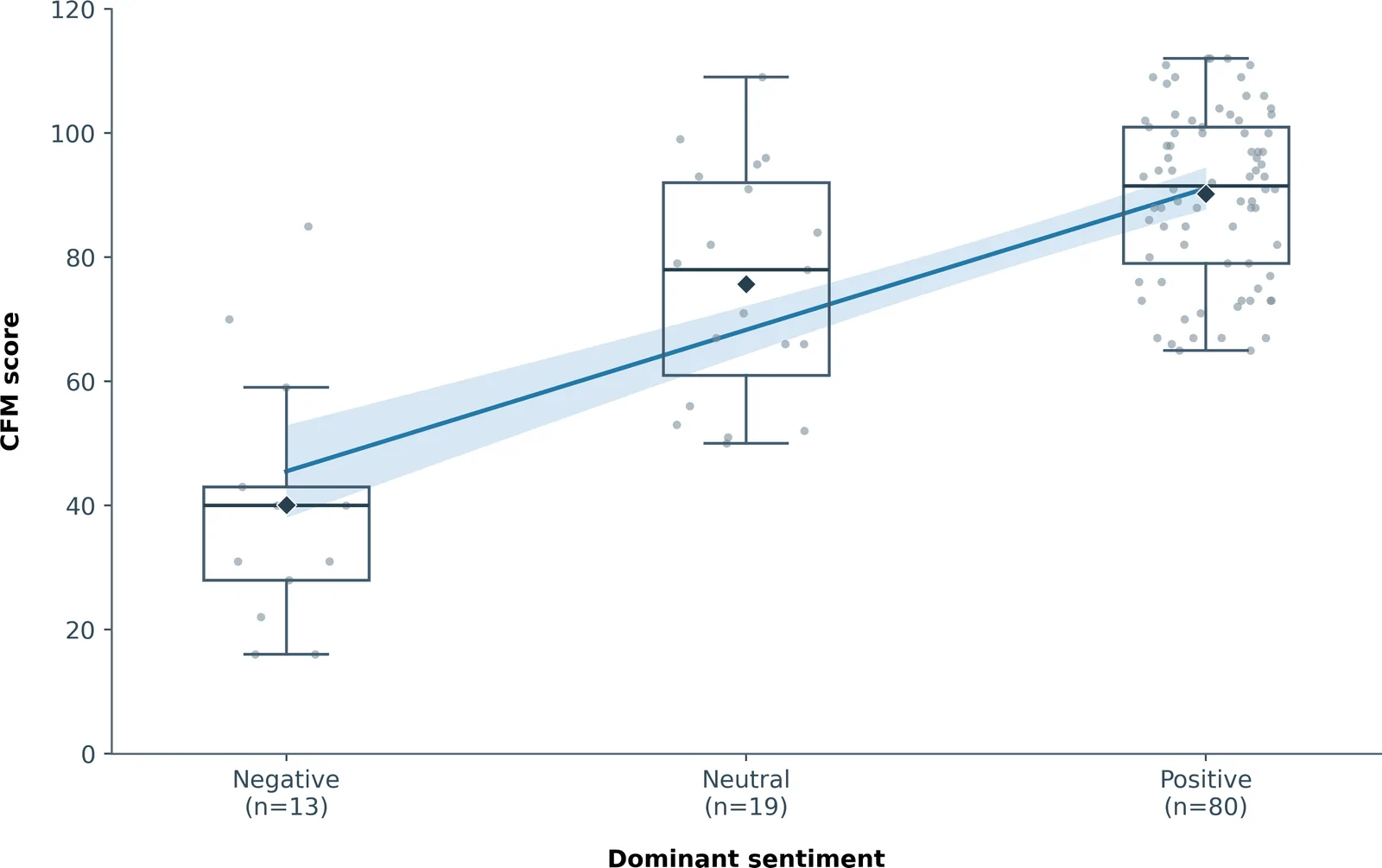
Linear trend in Couple Flourishing Measure (CFM) scores across dominant sentiment categories. Dominant sentiment was determined by the highest predicted probability generated by Pysentimiento for each narrative. Sentiment categories were coded as ordered levels (1=negative, 2=neutral, and 3=positive). The blue line represents the fitted linear regression, and the shaded area indicates the 95% CI for the estimated mean. Boxes represent IQRs, horizontal lines indicate medians, black diamonds represent group means, and gray points represent individual observations. Group sample sizes are displayed below each category.

### Qualitative Transformer Model Outputs

Given that Pysentimiento outperformed the lexical approach, narrative examples were included to illustrate how the model infers emotional probabilities from real descriptions of romantic relationships (Table 2). These examples show greater sensitivity to affective nuances, contextual recognition of emotional tone, and consistency between discursive meanings and assigned probabilities.

**Table 2.** Illustrative narratives with sentiment and emotion scores generated by Pysentimiento^a^.

Narrative category^b^	Examples of narratives	Negative	Positive	Neutral	Joy	Sadness	Anger	Disgust	Fear	Surprise
Negative narratives	“I consider it a toxic relationship, since I am never completely comfortable and I am in a vicious circle of reproaches that lead nowhere.”	0.982	0.002	0.016	0.005	0.060	0.777	0.063	0.003	0.001
Neutral narratives	“A contract that clearly sets out the terms of the relationship for both parties (what is tolerated and accepted, and what is not, what is expected from the relationship, specific behaviors, etc). The contract is modifiable, and clauses can be added as the relationship progresses. If either party does not accept the modifications, the contract is terminated, and the relationship ends.”	0.131	0.058	0.811	0.001	0.002	0.001	0.000	0.001	0.001
Positive narratives	“My relationship with my partner is very good; I have been married for many years and feel stable and very happy. It is one of the best things that has ever happened to me.”	0.003	0.972	0.025	0.970	0.001	0.000	0.001	0.001	0.001

a Values represent the predicted probabilities generated by Pysentimiento for each sentiment (negative, positive, and neutral) and emotion (joy, sadness, anger, disgust, fear, and surprise) category. Sentiment and emotion probabilities were obtained from separate classification tasks.

b Narrative excerpts are grouped according to their dominant sentiment classification.

## Discussion

### Principal Findings

The purpose of this study was to conduct a methodological comparison between 2 approaches to NLP: a lexicon-based model (NRC Emotion Lexicon) and a transformer-based model (Pysentimiento). Specifically, we examined the affective indices generated by each approach from spontaneous narratives about affective-sexual relationships and how these outputs relate to couple flourishing, as measured by the CFM.

The results show systematic differences between the 2 approaches. The NRC lexical model identified small but significant negative associations between CFM scores and anger, disgust, fear, sadness, and negative sentiment. These results are consistent with previous literature indicating that dictionary-based approaches can detect general affective trends but have significant limitations in capturing relational nuances, contextual ambiguity, polysemy, or implicit affective expressions [9,10,13]. This coincides with previous observations that point to the inherent limitations of lexicon-based models in emotionally complex and intimate domains [11,12].

In contrast, the transformer model (Pysentimiento) showed stronger associations with CFM scores across several affective dimensions. These associations suggest that Pysentimiento-derived indices may better reflect aspects of relational well-being captured by the CFM. Although this pattern is consistent with the potential advantages of contextual language representations, the present study did not directly assess the models’ ability to interpret linguistic nuance. These findings are consistent with recent studies that highlight the ability of deep learning–based models to represent complex affective patterns in natural languages [4,6,14]. This suggests that transformer-based approaches may offer methodological advantages when examining affective patterns in relational narratives.

From a theoretical and methodological perspective, these results are particularly relevant for the study of sexual-affective bonds. Narratives of this type of bond are often laden with metaphors, implicit meanings, relational nuances, and affective constructions that can be challenging for lexicon-based approaches to interpret. The stronger criterion-related associations observed for Pysentimiento suggest that transformer-based approaches may provide useful indicators of affective patterns associated with couple flourishing.

The observed linear trend, whereby CFM scores increased across the ordered dominant sentiment categories, provides additional evidence of an association between Pysentimiento-derived sentiment classifications and couple flourishing [26]. These findings contribute to the growing body of research on criterion-related evaluation of computational emotion detection in relational research.

Taken together, these findings highlight the potential of integrating NLP tools into research on sexual-affective relationships and, following further validation, into clinical applications. Transformer models may complement traditional self-report measures and offer alternative ways to study affective processes using natural discourse data, thus expanding theoretical and methodological possibilities in this field [27,28]. The comparison suggests that different NLP approaches may vary in their suitability for relational contexts, with contextual language processing representing a potentially relevant methodological advantage when examining affective meanings in intimate narratives.

In this context, these findings are particularly relevant to future research on clinical processes in couples therapy. Automated discourse analysis may provide additional information about affective signals that emerge spontaneously in interactions, which have traditionally been difficult to capture through manual coding or retrospective self-report measures [27]. Transformer-based models could be investigated for their potential to identify patterns of emotional communication associated with relational satisfaction or distress; detect moment-to-moment affective shifts during the process (ie, process-level markers); evaluate mechanisms of change, such as dyadic regulation, emotional responsiveness, or the emergence of shared narratives; and monitor therapeutic progress over time, potentially complementing traditional clinical assessments with context-sensitive indicators. However, these applications require direct empirical validation in therapeutic settings. These possibilities are consistent with recent advancements in psychotherapy-oriented NLP, in which sentiment-analysis models have been used to examine clients’ emotional expression across sessions [29] and to predict clinically relevant variables such as symptom severity [30]. Such potential applications are also consistent with findings by Steinbrenner et al [8], who demonstrated that NLP applied to psychotherapy transcripts could predict anxiety severity, suggesting possible applications for monitoring in therapeutic contexts. These contributions coincide with recent proposals suggesting that NLP innovations can deepen the understanding of therapeutic processes and open new avenues for analyzing relational change [34].

### Limitations and Future Directions

This study had several limitations. First, the sample consisted exclusively of participants residing in Spain, which limits the generalizability of the findings to other linguistic and cultural contexts. Second, although the transformer model showed stronger associations with CFM scores across several affective dimensions, these associations varied across emotion categories, indicating that the observed advantages were not uniform. This variability is consistent with challenges in computational emotion analysis previously discussed in the NLP literature [6]. Third, the cross-sectional design prevents conclusions about causal relationships between emotional discourse patterns and relational well-being. Fourth, the comparison was limited to 1 lexicon-based approach and 1 transformer-based model, restricting the generalizability of the findings to other tools within these computational paradigms. Fifth, multiple statistical comparisons were conducted without adjustment for multiple testing, increasing the possibility of type I errors. Therefore, individual statistically significant findings should be interpreted cautiously and require replication in independent samples. Finally, the analysis relied on a single open-ended narrative per participant and used CFM scores as an external criterion rather than independently annotated emotion labels. Consequently, the findings provide evidence of criterion-related associations but do not establish the accuracy of emotion classification. Furthermore, the use of nonprobability convenience sampling and self-reported data may have introduced selection and response biases, which should be considered when interpreting the findings.

Future research should extend the comparison to other languages, populations, and various types of sexual-affective relationships, as well as explore the integration of transformer models with qualitative methodologies or longitudinal designs to examine the evolution of emotional discourse in couples over time and assess the temporal reliability of model-derived indices. It would also be relevant to evaluate the use of these tools in clinical or intervention contexts, carefully considering ethical aspects related to privacy and the analysis of sensitive narratives. Furthermore, future research should evaluate how these models can be integrated into the observational analysis of therapeutic sessions to study the interactional microprocesses and mechanisms of change throughout treatment.

### Conclusions

This methodological comparison found that Pysentimiento showed stronger associations with couple flourishing than the NRC Emotion Lexicon across several affective dimensions, although these differences were not consistent across all categories. These results contribute to methodological advances in the study of romantic and sexual relationships by highlighting the value of theory-driven external criteria for evaluating computational emotion detection and open new possibilities for the automated analysis of emotional discourse in relational contexts. Furthermore, they suggest promising directions for research on processes in couples therapy, highlighting the potential of NLP as a complementary tool in clinical research on relational processes, subject to further empirical validation.

## Abbreviations

CFM: Couple Flourishing Measure
ESF+: European Social Fund Plus
NLP: natural language processing
